# Supplementary figures and images for: A classification framework for Bacillus anthracis defined by global genomic structure
Source: Evol Appl. 2020 Jan 23;13(5):935–44. doi: 10.1111/eva.12911 (PMC7232756; doi:10.1111/eva.12911)

(a)

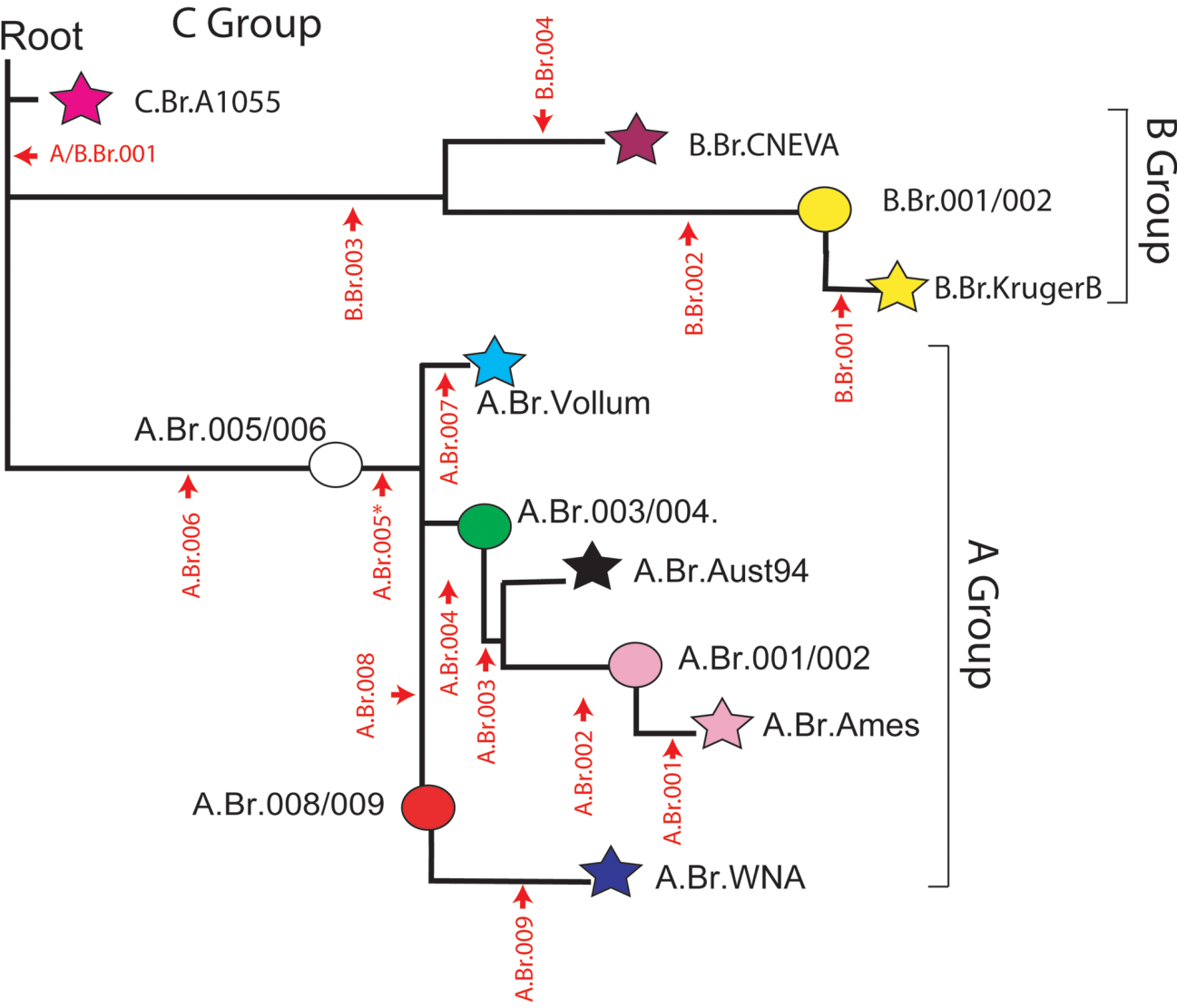

(b)

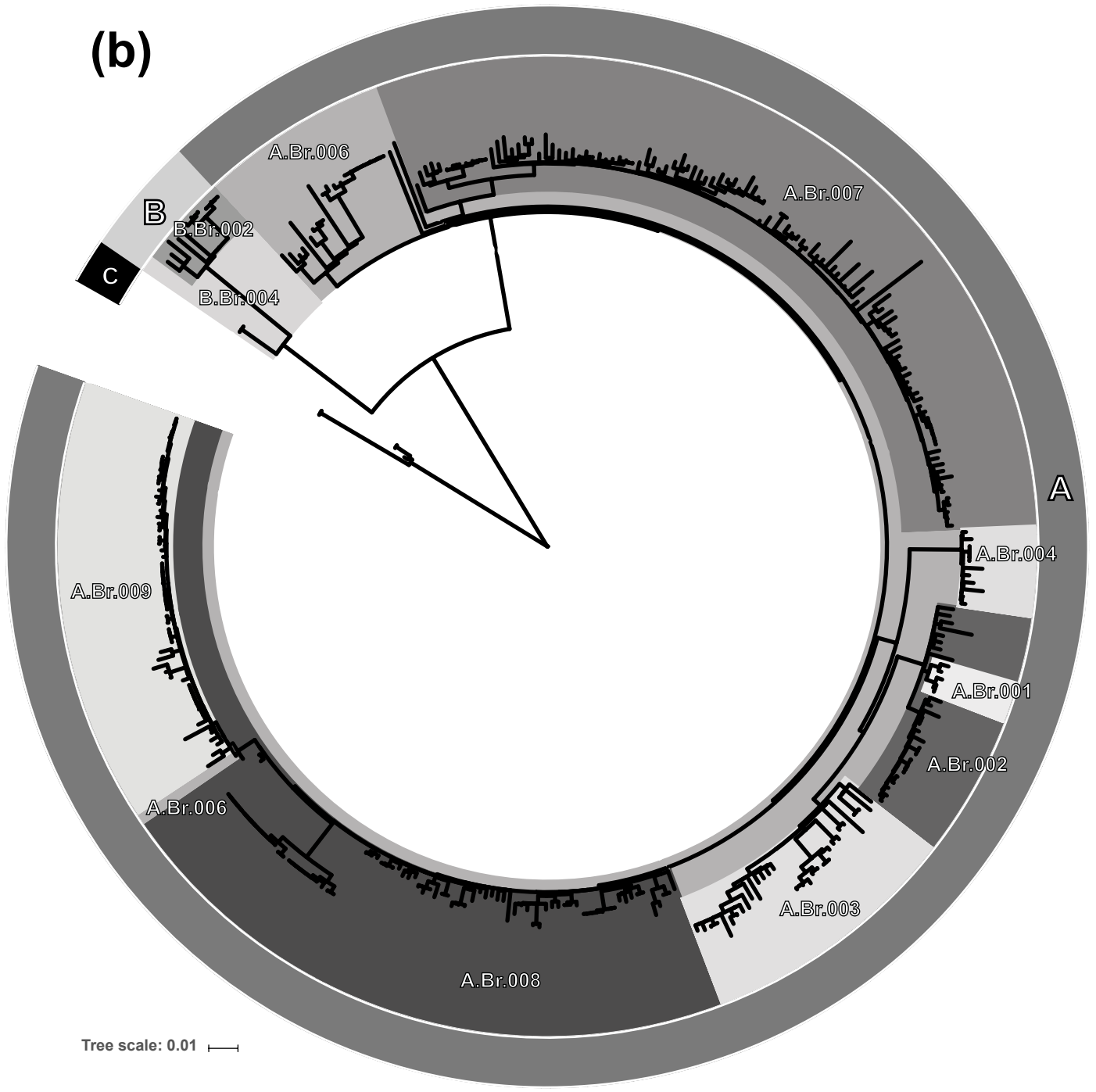

Supplement: Supplementary file 1 [file EVA-13-935-s001.pdf]
