# Supplementary material for: A classification framework for Bacillus anthracis defined by global genomic structure
Source: Evol Appl. 2020 Jan 23;13(5):935–44. doi: 10.1111/eva.12911 (PMC7232756; doi:10.1111/eva.12911)
Supplement: Supplementary file 2 [file EVA-13-935-s002.docx]

**
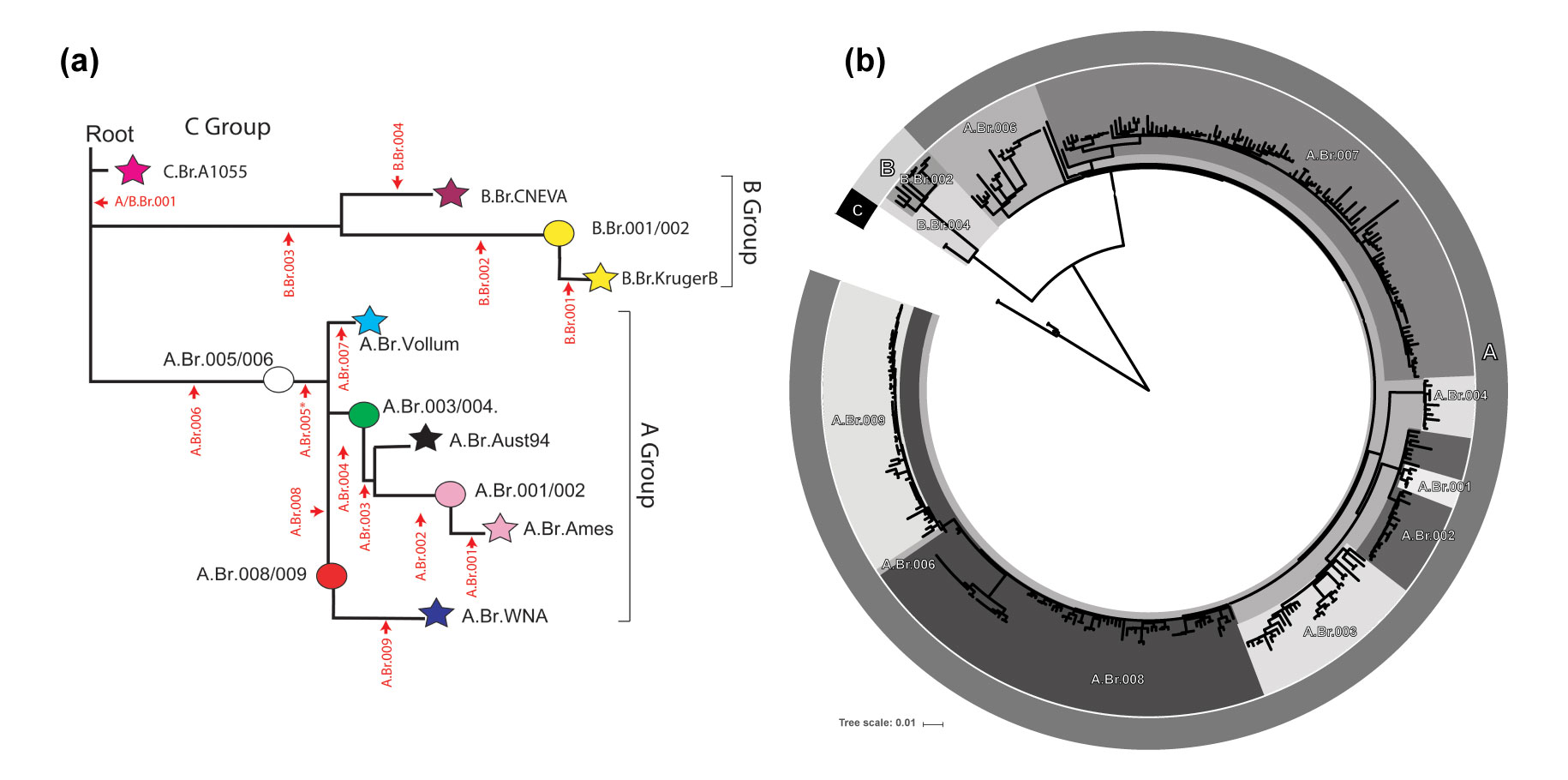
**

**S1 Fig. Chromosomal phylogeny for the *B. anthracis* global dataset in relation to the Van Ert *et al.* classification scheme.** (a) The relationship between canSNPs, sub-lineages and/or sub-groups from Van Ert et al., 2007: The stars in this dendrogram represent specific lineages that are defined by one of the seven sequenced genomes of *B. anthracis*. The circles represent branch points along the lineages that contain specific subgroups of isolates. These sub-groups are named after the canSNPs that flank these positions. Indicated in red are the positions and names for each of the canSNPs (b) Whole-chromosome tree of 356 global *B. anthracis* isolates shaded and labeled using the genotyping scheme shown in a*.* De novo assemblies were performed on paired raw read files using SPAdes, and classified with the CanSNPer package. All tree annotation was carried out using iTOL.

**S1 Table**

| Group | N | In | Out | Position | Locus | Gene |
| --- | --- | --- | --- | --- | --- | --- |
| 1.1 | 2 | A | T | 1771938 | GBAA_1887 | enterotoxin A |
| 1.2^a^ | 3 | T | C | 1201909 | GBAA_1246 | sodium/proline symporter family protein |
| 2.1 | 2 | A | G | 2212885 | GBAA_2372 | dhbf |
| 2.2^b^ | 5 | G | A | 2132895 | GBAA_2284 | hypothetical protein |
| 2.3^c^ | 6 | A | G | 2019941 | GBAA_2169 | transcriptional regulator, TetR family |
| 3.1 | 6 | C | A | 3086718 | GBAA_3355 | alo |
| 3.2^d^ | 4 | C | T | 3366432 | GBAA_3657 | parE |
| 3.3^d^ | 11 | T | G | 3359543 | GBAA_3652 | gene |
| 4.1 | 12 | C | T | 810017 | GBAA_0794 | hypothetical protein |
| 4.2^e^ | 63 | T | C | 837700 | GBAA_0825 | conserved hypothetical protein |
| 4.3^f^ | 35 | G | A | 1271875 | GBAA_1327 | phaP protein |
| 5.1 | 11 | T | C | 857338 | GBAA_0848 | conserved hypothetical protein |
| 5.2^g^ | 29 | C | A | 4499321 | GBAA_4954 | hypothetical protein |
| 5.3^g^ | 24 | T | C | 569295 | GBAA_0561 | citrate transporter, CitM family |
| 5.4^h^ | 8 | T | C | 565120 | GBAA_0558 | methyl-accepting chemotaxis protein |
| 6.1 | 57 | T | C | 2332200 | GBAA_2510 | csbX |
| 6.2^i^ | 22 | T | C | 2387683 | GBAA_2566 | acetyltransferase, GNAT family |
| 6.3^i^ | 56 | A | G | 1776654 | GBAA_1891 | putative deoxyribonucleoside regulator DeoR |

**S1 Table. Canonical SNPs for classifying *B. anthracis* isolates.** Key: Group = Primary cluster/Clade; N = number of *B. anthracis* isolates in the Group; In = allele within the group; Out = allele outside of group; ^a^ nested in 1.1; ^b^ nested in 2.1; ^c^ nested in 2.2; ^d^ nested in 3.1; ^e^ nested in 4.1; ^f^ nested in 4.2; ^g^ nested in 5.1; ^h^ nested in 5.3; ^i^ nested in 6.1.

**S2 Table**

| Accession | Origin | Isolation date | Failed bacterial component |
| --- | --- | --- | --- |
| SRR5810989 | Argentina | 1964 | pXO2 |
| SRR5811192 | Argentina | 1964 | pXO1, pXO2 |
| SRR2968170 | Botswana | - | pXO2 |
| SRR2339291 | USA: California | 1976 | Chromosome, pXO1, pXO2 |
| SRR2339617 | USA: California | 1976 | Chromosome, pXO1, pXO2 |
| SRR2340465 | USA: California | 1976 | Chromosome, pXO1, pXO2 |
| SRR5811182 | USA: California | 1976 | pXO2 |
| SRR5811208 | USA: California | 1976 | pXO1, pXO2 |
| SRR5811191 | Canada | 1963 | pXO1, pXO2 |
| SRR5811247 | Canada | 1963 | pXO1 |
| SRR2157175 | USA: Florida | 1951 | pXO2 |
| SRR2339516 | Haiti | 1976 | Chromosome, pXO1, pXO2 |
| SRR2339619 | Haiti | 1976 | Chromosome, pXO1, pXO2 |
| SRR2340858 | Haiti | 1977 | Chromosome, pXO1, pXO2 |
| SRR5811210 | Haiti | 1977 | pXO1 |
| SRR2340466 | Haiti | 1981 | Chromosome, pXO1, pXO2 |
| SRR2340467 | USA: Iowa | 1979 | Chromosome, pXO1, pXO2 |
| SRR3418280 | Kyrgyzstan | 2011 | pXO2 |
| SRR3418281 | Kyrgyzstan | 2011 | pXO2 |
| SRR3418282 | Kyrgyzstan | 2011 | pXO2 |
| SRR3418292 | Kyrgyzstan | 2011 | pXO2 |
| SRR5811162 | USA: Louisiana | 1956 | pXO1, pXO2 |
| SRR5947105 | USA: Louisiana | 1956 | pXO1 |
| SRR2145877 | USA: Louisiana | - | pXO1 |
| SRR2145878 | USA: Louisiana | - | Chromosome, pXO1, pXO2 |
| SRR5811216 | USA: Maryland | 1960 | pXO1, pXO2 |
| SRR5810964 | USA: Massachusetts | 1974 | pXO1, pXO2 |
| SRR5810965 | USA: Massachusetts | 1974 | pXO2 |
| SRR5811041 | USA: Massachusetts | 1974 | pXO1, pXO2 |
| SRR2340910 | USA: Massachusetts | 1976 | Chromosome, pXO1, pXO2 |
| SRR5810983 | USA: Mississippi | 1957 | pXO1 |
| SRR5811070 | USA: Mississippi | 1962 | pXO1 |
| SRR5811168 | USA: Mississippi | 1962 | pXO1, pXO2 |
| SRR5810951 | USA: New Hampshire | 1958 | pXO1 |
| SRR5810952 | USA: New Hampshire | 1958 | pXO1, pXO2 |
| SRR5810953 | USA: New Hampshire | 1958 | pXO2 |
| SRR5810954 | USA: New Hampshire | 1958 | pXO1, pXO2 |
| SRR5810991 | USA: New Hampshire | 1958 | pXO2 |
| SRR5811248 | USA: New Hampshire | 1958 | pXO1, pXO2 |
| SRR5811099 | USA: New Hampshire | 1959 | pXO2 |
| SRR5811100 | USA: New Hampshire | 1959 | pXO2 |
| SRR2339640 | USA: New Hampshire | 1978 | Chromosome, pXO1, pXO2 |
| SRR2339645 | USA: New Hampshire | 1978 | Chromosome, pXO1, pXO2 |
| SRR2340010 | USA: New Hampshire | 1978 | Chromosome, pXO1, pXO2 |
| SRR2340073 | USA: New Hampshire | 1978 | Chromosome, pXO1, pXO2 |
| SRR2340171 | USA: New Hampshire | 1978 | Chromosome, pXO1, pXO2 |
| SRR2340230 | USA: New Hampshire | 1978 | Chromosome, pXO1, pXO2 |
| SRR2340486 | USA: New Hampshire | 1978 | Chromosome, pXO1, pXO2 |
| SRR2340857 | USA: New Hampshire | 1978 | Chromosome, pXO1, pXO2 |
| SRR5810966 | USA: New Hampshire | 1978 | pXO1 |
| SRR5810967 | USA: New Hampshire | 1978 | pXO2 |
| SRR5810968 | USA: New Hampshire | 1978 | pXO1, pXO2 |
| SRR5810969 | USA: New Hampshire | 1978 | pXO2 |
| SRR5810970 | USA: New Hampshire | 1978 | pXO1, pXO2 |
| SRR5810971 | USA: New Hampshire | 1978 | pXO1, pXO2 |
| SRR5810972 | USA: New Hampshire | 1978 | pXO2 |
| SRR5811006 | USA: New Hampshire | 1978 | pXO1, pXO2 |
| SRR5811147 | USA: New Hampshire | 1978 | pXO2 |
| SRR5811230 | USA: New Hampshire | 1978 | pXO2 |
| SRR5811234 | USA: New Hampshire | 1978 | pXO1, pXO2 |
| SRR2340305 | USA: New Jersey | 1975 | Chromosome, pXO1, pXO2 |
| SRR5810962 | USA: New Jersey | 1960 | pXO1, pXO2 |
| SRR2340252 | USA: New Jersey | 1975 | pXO1 |
| SRR5811079 | USA: New Jersey | 1975 | pXO1, pXO2 |
| SRR5811124 | USA: New Jersey | 1975 | pXO1, pXO2 |
| SRR5811125 | USA: New Jersey | 1975 | pXO1 |
| SRR5811126 | USA: New Jersey | 1975 | Chromosome, pXO1, pXO2 |
| SRR2340461 | USA: New Jersey | 1976 | Chromosome, pXO1, pXO2 |
| SRR2340463 | USA: New Jersey | 1976 | Chromosome, pXO1, pXO2 |
| SRR2340464 | USA: New Jersey | 1976 | Chromosome, pXO1, pXO2 |
| SRR2895370 | USA: New Jersey | 1976 | Chromosome, pXO1, pXO2 |
| SRR5811181 | USA: New Jersey | 1976 | pXO1, pXO2 |
| SRR5811184 | USA: New Jersey | 1976 | pXO1, pXO2 |
| SRR5811185 | USA: New Jersey | 1976 | pXO1, pXO2 |
| SRR5811225 | USA: New Jersey | 1976 | pXO2 |
| SRR5811209 | USA: New York | 1976 | pXO1 |
| SRR5811005 | USA: North Carolina | 1957 | pXO1, pXO2 |
| SRR5811061 | USA: North Carolina | 1957 | pXO1, pXO2 |
| SRR5811050 | USA: North Carolina | 1961 | pXO1 |
| SRR2339241 | USA: North Carolina | 1978 | Chromosome, pXO1, pXO2 |
| SRR2340468 | USA: North Carolina | 1978 | Chromosome, pXO1, pXO2 |
| SRR2340469 | USA: North Carolina | 1978 | Chromosome, pXO1, pXO2 |
| SRR2340470 | USA: North Carolina | 1978 | Chromosome, pXO1, pXO2 |
| SRR2340471 | USA: North Carolina | 1978 | Chromosome, pXO1, pXO2 |
| SRR2340474 | USA: North Carolina | 1978 | Chromosome, pXO1, pXO2 |
| SRR2340475 | USA: North Carolina | 1978 | Chromosome, pXO1, pXO2 |
| SRR2340476 | USA: North Carolina | 1978 | Chromosome, pXO1, pXO2 |
| SRR2340477 | USA: North Carolina | 1978 | Chromosome, pXO1, pXO2 |
| SRR2340478 | USA: North Carolina | 1978 | Chromosome, pXO1, pXO2 |
| SRR2340480 | USA: North Carolina | 1978 | Chromosome, pXO1, pXO2 |
| SRR2340481 | USA: North Carolina | 1978 | Chromosome, pXO1, pXO2 |
| SRR2340482 | USA: North Carolina | 1978 | Chromosome, pXO1, pXO2 |
| SRR2340483 | USA: North Carolina | 1978 | Chromosome, pXO1, pXO2 |
| SRR2340485 | USA: North Carolina | 1978 | Chromosome, pXO1, pXO2 |
| SRR2340487 | USA: North Carolina | 1978 | Chromosome, pXO1, pXO2 |
| SRR2340488 | USA: North Carolina | 1978 | Chromosome, pXO1, pXO2 |
| SRR2340490 | USA: North Carolina | 1978 | Chromosome, pXO1, pXO2 |
| SRR2340492 | USA: North Carolina | 1978 | Chromosome, pXO1, pXO2 |
| SRR2340505 | USA: North Carolina | 1978 | Chromosome, pXO1, pXO2 |
| SRR2340670 | USA: North Carolina | 1978 | Chromosome, pXO1, pXO2 |
| SRR2340697 | USA: North Carolina | 1978 | Chromosome, pXO1, pXO2 |
| SRR2340730 | USA: North Carolina | 1978 | Chromosome, pXO1, pXO2 |
| SRR2340969 | USA: North Carolina | 1978 | Chromosome, pXO1, pXO2 |
| SRR2340986 | USA: North Carolina | 1978 | Chromosome, pXO1, pXO2 |
| SRR5811016 | USA: North Carolina | 1978 | pXO1, pXO2 |
| SRR5811017 | USA: North Carolina | 1978 | pXO1 |
| SRR5811019 | USA: North Carolina | 1978 | pXO1, pXO2 |
| SRR5811020 | USA: North Carolina | 1978 | pXO1 |
| SRR5811021 | USA: North Carolina | 1978 | pXO1 |
| SRR5811121 | USA: North Carolina | 1978 | pXO1 |
| SRR5811122 | USA: North Carolina | 1978 | pXO1 |
| SRR5811128 | USA: North Carolina | 1978 | pXO1, pXO2 |
| SRR5811129 | USA: North Carolina | 1978 | pXO2 |
| SRR5811178 | USA: North Carolina | 1978 | pXO1, pXO2 |
| SRR5811205 | USA: North Carolina | 1978 | pXO1 |
| SRR5811228 | USA: North Carolina | 1978 | pXO1, pXO2 |
| SRR5811046 | USA: North Carolina | 1987 | pXO2 |
| SRR5811049 | USA: North Carolina | 1987 | pXO2 |
| SRR2339961 | USA: North Carolina | 1988 | Chromosome, pXO1, pXO2 |
| SRR5811048 | USA: North Carolina | 1988 | pXO1 |
| DRR128188 | Japan: Okayama |  | Chromosome, pXO2 |
| SRR5811167 | Pakistan | 1961 | pXO2 |
| SRR2339632 | Paraguay | - | Chromosome, pXO1, pXO2 |
| SRR2339823 | USA: Pennsylvania | 1974 | Chromosome, pXO1, pXO2 |
| SRR5811083 | USA: Pennsylvania | 1974 | pXO1, pXO2 |
| SRR2339757 | USA: Rhode Island | 1974 | Chromosome, pXO1, pXO2 |
| SRR2339940 | USA: Rhode Island | 1974 | Chromosome, pXO1, pXO2 |
| SRR5811213 | USA: Rhode Island | 1974 | pXO1 |
| SRR2968141 | Russia | 1979 | Chromosome |
| SRR2968143 | Russia | 1979 | Chromosome |
| SRR2968198 | Russia | 1979 | Chromosome, pXO1, pXO2 |
| SRR2968216 | Russia | 1979 | Chromosome, pXO1, pXO2 |
| SRR2968168 | Russia | - | pXO2 |
| SRR2968169 | Russia | - | pXO2 |
| SRR5811076 | South Africa | 1961 | pXO2 |
| SRR2339898 | South Africa | 1974 | Chromosome, pXO1, pXO2 |
| SRR5811080 | South Africa | 1974 | pXO2 |
| SRR5811082 | South Africa | 1974 | pXO1, pXO2 |
| SRR5811085 | South Africa | 1974 | pXO1 |
| SRR5811215 | South Africa | 1974 | pXO1 |
| SRR1739968 | South Africa | - | pXO2 |
| SRR5811037 | South Africa | - | pXO2 |
| SRR5810963 | USA: South Carolina | 1956 | pXO1, pXO2 |
| SRR5811003 | USA: South Carolina | 1956 | pXO1, pXO2 |
| SRR2339626 | USA: South Carolina | 1960 | Chromosome, pXO1, pXO2 |
| SRR5811063 | USA: South Carolina | 1960 | pXO1, pXO2 |
| SRR5810959 | USA: South Carolina | 1961 | pXO1, pXO2 |
| SRR5811004 | USA: South Carolina | 1961 | pXO1, pXO2 |
| SRR5810997 | USA: South Carolina | 1963 | pXO1, pXO2 |
| SRR5811000 | USA: South Carolina | 1963 | pXO2 |
| SRR5811007 | USA: South Carolina | 1963 | pXO1, pXO2 |
| SRR5810998 | USA: South Carolina | 1964 | pXO1, pXO2 |
| SRR5811069 | USA: Texas | 1976 | pXO1, pXO2 |
| SRR5811166 | USA: Texas | 1976 | pXO1 |
| SRR5811218 | USA: Texas | 1976 | pXO1 |
| SRR2339631 | USA: Texas | 1988 | Chromosome, pXO1, pXO2 |
| SRR5811145 | USA: Texas | 1988 | pXO2 |
| SRR5811146 | USA: Texas | 1988 | pXO2 |
| SRR5811219 | Thailand | 1964 | pXO1, pXO2 |
| SRR5811187 | Thailand | 1965 | pXO1 |
| SRR2968136 | Turkey | - | pXO2 |
| SRR2968144 | Turkey | - | pXO1 |
| SRR2968193 | Turkey | - | pXO2 |
| SRR2340304 | United Kingdom | 1975 | Chromosome, pXO1, pXO2 |
| SRR5811120 | United Kingdom | 1975 | pXO2 |
| SRR5811123 | United Kingdom | 1975 | pXO1, pXO2 |
| SRR2094267 | United Kingdom | 2006 | pXO2 |
| SRR2094270 | United Kingdom | 2006 | pXO2 |
| SRR2094273 | United Kingdom | 2006 | pXO2 |
| SRR5811034 | United States | 1956 | pXO2 |
| SRR5811101 | United States | 1956 | pXO1, pXO2 |
| SRR5811103 | United States | 1956 | pXO1, pXO2 |
| SRR5811104 | United States | 1956 | pXO1, pXO2 |
| SRR2340460 | USA: Utah | 1975 | Chromosome, pXO1, pXO2 |
| SRR5811084 | USA: Utah | 1975 | pXO1, pXO2 |
| SRR2339728 | USA: Washington | 1974 | Chromosome, pXO1, pXO2 |
| SRR5811040 | USA: Washington | 1974 | pXO1, pXO2 |
| SRR5811078 | USA: Washington | 1974 | pXO2 |
| SRR5811081 | USA: Washington | 1974 | pXO1, pXO2 |
| SRR5811086 | USA: Washington | 1974 | pXO2 |
| SRR5811190 | USA: Washington | 1974 | pXO2 |
| SRR1739967 | USA: Wyoming | 1956 | pXO1 |
| SRR5811161 | USA: Wyoming | 1956 | pXO1, pXO2 |
| SRR5811214 | USA: Wyoming | 1956 | pXO1 |
| SRR5811096 | USA: Wyoming | 1961 | pXO1 |
| DRR014738 | Zambia | 2013 | Chromosome, pXO1, pXO2 |

**S2 Table. Isolate information for individuals that failed to meet coverage and read depth criteria**. Isolates listed below failed to meet coverage and read depth criteria (see methods) when mapped to the Ames Ancestor reference genome (accession number GCA_000008445.1; Chromosome: AE017334, pXO1 replicon: AE017336, pXO2 replicon: AE017335).

**S1 Dataset. Metadata for all isolates included in this study.** This excel file includes strain information, NCBI accession numbers, years of isolation (if known), continent, country/region of origin, isolation source, genotype classification and submission source information for all isolates.

**S1 File. Python script for rapid *B. anthracis* genotyping.** This script can be used for rapid primary cluster and clade designation using *B. anthracis* draft genomes (mapped to a reference) or mapped raw read data in the form of BAM or VCF files.
